# Association of Cbl-Interacting Protein CIN85 with Protein Phosphatase 2A (PP2A) Partly Confer Neoplastic Properties to Human Leukemia Cell Lines

**DOI:** 10.3390/cells15201897

**Published:** 2026-10-19

**Authors:** Malak Abdullah Aldahami, Rawan Abdulrahman Alfrayh, Yasser Basmaeil, Haya Nasser Bin Kulayb, Sabiha Fatima, Bader Alahmari, Tanvir Khatlani

**Affiliations:** 1 Stem Cells and Regenerative Medicine, Blood and Cancer Research Department (BCR), King Abdullah International Medical Research Center (KAIMRC), King Saud Bin Abdulaziz University for Health Sciences (KSAU), King Abdulaziz Medical City, Ministry of National Guard Health Affairs (MNGHA), Riyadh 11426, Saudi Arabia; malak.aldahami@gmail.com (M.A.A.); ralfrayh@ksu.edu.sa (R.A.A.); basmaeily@kaimrc.med.sa (Y.B.); binkulaybh@kaimrc.med.sa (H.N.B.K.); ahmarib@kaimrc.med.sa (B.A.); 2 Department of Biochemistry, College of Sciences, King Saud University, Riyadh 11451, Saudi Arabia; 3 Department of Clinical Laboratory Sciences, College of Applied Medical Sciences, King Saud University, Riyadh 11451, Saudi Arabia; sabmehdi@ksu.edu.sa

**Keywords:** protein phosphatase 2A, CIN85, leukemia, phosphatase activating drugs (PADs), proliferation

## Abstract

**Highlights:**

**What are the main findings?**

- In leukemia, Protein phosphatase PP2A expression and activity is downregulated. CIN85, an adaptor protein is highly expressed and it interacts with PP2A blocking its normal functioning.
- CIN85 in relation to PP2Ac has not been previously known to play a role in leukemia. This makes it a completely new target for designing future cancer treatments.

**What are the implications of the main findings?**

- Physical blocking the CIN85 interaction with PP2A leads to its release from the complex, resulting in diminished growth of cancer cells.
- Targeting CIN85-PP2A complex using genetic and pharmacological approach will open new areas for therapy against leukemia.

**Abstract:**

Protein phosphatase 2A (PP2A) is a critical tumor suppressor frequently inactivated in hematological malignancies, yet the mechanisms underlying this dysregulation remain poorly defined. Here, we investigated a novel interaction between the PP2A catalytic subunit (PP2Ac) and the adaptor protein CIN85. Analyzing transcriptomic datasets from AML, CML, CLL, and ALL patients, alongside functional validation in K562 and THP1 leukemia cell lines via RT-qPCR, immunoblotting, phosphatase activity assays, pharmacological modulation (FTY720 and Okadaic acid), siRNA-mediated knockdown, and proliferation assays, we found that CML and CLL exhibit reduced PP2A and elevated CIN85, with activated AKT, SRC, and GSK-3β levels. Pharmacological activation of PP2A reduced AKT and GSK-3β phosphorylation, while inhibition had opposing effects, confirming PP2A’s regulatory role. Crucially, CIN85 silencing markedly enhanced PP2A phosphatase activity without changing PP2Ac protein levels, leading to robust dephosphorylation of AKT, SRC, and GSK-3β and significantly suppressing leukemic cell proliferation. Collectively, these results identify CIN85 as a previously unrecognized endogenous negative regulator that directly suppresses PP2A function in leukemia. Importantly, disrupting the PP2Ac-CIN85 complex effectively restores PP2A tumor-suppressive activity, dampens oncogenic signaling, and inhibits growth, providing mechanistic clarity and positioning this protein-protein interface as one of the possible therapeutic targets for treating hematological cancers.

## 1. Introduction

Reversible protein phosphorylation is one of the most tightly regulated post-translational modifications in eukaryotic cells and serves as a fundamental mechanism for controlling cellular signaling. The phosphorylation status of proteins determines their functional properties, including enzymatic activity, protein stability, subcellular localization, and protein-protein interactions [1]. Under physiological conditions, protein kinases and phosphatases function in concert to maintain phosphorylation homeostasis by regulating the phosphorylation state of target proteins [2]. This dynamic balance is essential for normal cellular function, and its disruption is a hallmark of many human malignancies, particularly hematological cancers. Although dysregulated protein kinases have been extensively implicated in malignant transformation, comparatively less attention has been given to the role of protein phosphatases in cancer development and leukemogenesis [3,4].

Among serine/threonine phosphatases, Protein Phosphatase 2A (PP2A) is one of the major intracellular phosphatases responsible for a large proportion of cellular dephosphorylation events. PP2A is a heterotrimeric holoenzyme composed of a catalytic (C; PP2Ac), a structural scaffolding (A; PP2Aa), and a variable regulatory (B; PP2Ab) subunit that determines substrate specificity, enzymatic activity, and subcellular localization [5,6,7]. Through dephosphorylation of multiple substrates, including AKT, MYC, BCR-ABL, and c-Src, PP2A regulates diverse cellular processes such as cell cycle progression, apoptosis, DNA damage repair, differentiation, and metabolism [8]. In addition, PP2A negatively regulates several major oncogenic signaling pathways, including PI3K/AKT/mTOR, WNT, JAK/STAT, NF-κB, and RAS/MAPK, which are frequently hyperactivated in cancer [7]. Consequently, PP2A is widely recognized as a critical tumor suppressor, although its biological functions remain highly context dependent.

Loss of PP2A activity contributes to the pathogenesis of numerous cancers through genetic alterations, post-translational modifications, or overexpression of endogenous inhibitory proteins, resulting in sustained activation of oncogenic signaling pathways [9,10]. In hematological malignancies, leukemogenesis frequently results from disruption of the balance between kinase and phosphatase activities, with functional suppression of PP2A permitting persistent activation of proliferative and survival signaling [9,10]. Functional inactivation of PP2A has been reported in chronic myeloid leukemia (CML) and acute myeloid leukemia (AML), primarily through overexpression of the endogenous inhibitors SET and CIP2A or loss-of-function mutations in scaffold or regulatory subunits [10,11]. Because PP2A is recurrently inactivated in leukemia, it has emerged as an attractive therapeutic target. Pharmacological PP2A-activating drugs (PADs) restore PP2A tumor-suppressive activity, suppress leukemic progression, and reduce leukemic stem cell burden, supporting PP2A reactivation as a promising therapeutic strategy for refractory leukemia [12].

Besides alterations in kinases and phosphatases, dysregulation of adaptor proteins represents another important mechanism driving aberrant signaling in cancer. Adaptor molecules, also known as scaffold proteins, are non-enzymatic proteins that facilitate signal transduction by assembling multi-protein signaling complexes [13]. Using specialized interaction domains such as Src homology 2 (SH2) and Src homology 3 (SH3), adaptor proteins coordinate signaling specificity, amplification, and integration. Consequently, altered adaptor protein expression or function contributes to uncontrolled proliferation, resistance to apoptosis, metastasis, and therapeutic resistance [14].

Adaptor proteins are broadly classified into cytoplasmic, transmembrane, and nuclear families. Among the cytoplasmic adaptor proteins, Cbl-interacting protein of 85 kDa (CIN85) is a multifunctional scaffold that interacts with numerous signaling proteins [15,16]. Through these interactions, CIN85 regulates receptor trafficking, intracellular signaling, cytoskeletal organization, and cell survival. Disruption of the c-Cbl-CIN85 interaction impairs degradation of BCR and receptor tyrosine kinases (RTKs), leading to sustained survival signaling in CML, AML, and acute lymphoblastic leukemia (ALL), thereby contributing to leukemogenesis [15,16,17,18].

Recently, we identified a previously unrecognized interaction between PP2Ac and CIN85. Our earlier study demonstrated that formation of the PP2Ac-CIN85 complex suppresses PP2A phosphatase activity in vitro. Moreover, pharmacological and genetic disruption of this complex restored PP2Ac activity and reduced phosphorylation of multiple oncogenic kinases, including AKT, Src, STAT3, and glycogen synthase kinase-3β (GSK-3β), suggesting that CIN85 functions as a negative regulator of PP2A signaling [19].

In this study, we investigated the biological significance of the PP2Ac-CIN85 interaction in hematological malignancies and examined the consequences of disrupting this complex using genetic approaches in leukemia cell lines. We found that PP2Ac is moderately expressed in primary human leukemia samples, whereas CIN85 is markedly overexpressed in both leukemia patient samples and leukemia-derived cell lines. Importantly, disruption of the PP2Ac-CIN85 complex significantly enhanced PP2A phosphatase activity, leading to dephosphorylation of hyperactivated kinases, including AKT and GSK-3β, and suppression of leukemia cell proliferation. Collectively, these findings identify the PP2Ac-CIN85 interaction as a previously underappreciated mechanism regulating PP2Ac activity and suggest that targeting this complex may represent a novel therapeutic strategy for restoring PP2A tumor-suppressive function in hematological malignancies.

## 2. Materials and Methods

### 2.1. Dataset Selection and Retrieval

Gene expression data from peripheral blood or bone marrow samples of leukemia patients and healthy controls were retrieved from the Gene Expression Omnibus (GEO) repository (https://www.ncbi.nlm.nih.gov/geo (accessed on 5 March 2026)). The search was conducted using the keywords “leukemia,” “Acute Myeloid Leukemia” (AML), “Chronic Myeloid Leukemia” (CML), “Chronic Lymphocytic Leukemia” (CLL), and “Acute Lymphocytic Leukemia” (ALL), along with “gene expression profiling”, “protein phosphatases”, “serine/threonine protein phosphatases”, and “adaptor proteins”. The inclusion criteria for datasets were: [1] contained both leukemia and normal control samples; and [2] had publicly available raw data (.CEL files) or normalized expression matrices.

Out of nine datasets, three datasets for CML, two datasets for AML, three datasets for CLL, and one dataset for ALL, was selected for this study. These included CML (GSE144119 with 16 patients and 6 controls, GSE40721 with 5 patients and 4 controls, and GSE407927 with 12 patients and 3 controls. For AML, GSE9476 with 26 patients and 38 normal and GSE43176 with 104 patients and 4 normal were selected. For CLL, the datasets were GSE22529 with 41 patients and 11 normal, GSE18026 with 5 patients and 2 normal, and GSE36907 with 20 patients and 45 normal. And for ALL, GSE28497, including 101 patients and 04 normal, was selected (Supplementary Table S1). The data were profiled on the Affymetrix Human Genome U133A and U133B Chips, Plus 2.0 Array, Catalog # 900471 (Applied Biosystems, Waltham, MA, USA).

### 2.2. Data Processing and Normalization

To analyze Affymetrix microarray data from the Gene Expression Omnibus (GEO), we used the online tool GEO2R. For Affymetrix data, normalization had already been performed by the data submitter by following the RMA pipeline, incorporating background correction, quantile normalization, and log^2^ transformation, allowing us to directly import the already normalized data. By default, GEO2R applies the Benjamini-Hochberg [20] False Discovery Rate method, converting raw *p*-values into adjusted q-values (adj.P.Val) to control for false positives, and it generates a results table containing logFC (log^2^ fold change), a moderated t-statistic, the raw *p*-value, the critical adjusted *p*-value (commonly filtered at <0.05 or <0.01), and the B-statistic, which reflects the log odds of differential expression. Since GEO2R outputs probe-level data, mapping of the same gene was handled by retaining the probe row with the highest statistical significance during post-processing.

The full results tables were then downloaded, imported into Excel, and filtered using the “Gene. Symbol” column to exclude blanks and non-genic control entries. To isolate differentially expressed genes, we applied statistical cutoffs by filtering “adj.P.Val” below 0.05 and retaining only rows where the absolute logFC exceeded 1, corresponding to a minimum two-fold change. Finally, the filtered list was separated into upregulated genes (logFC > 0) and downregulated genes (logFC < 0) by exporting them into distinct worksheets, thereby generating a clean, finalized DEG list.

### 2.3. Cell Lines and Reagents

Human leukemia cell lines K-562 (ATCC CCL-243) and THP-1 (ATCC TIB-202), as well as HEK-293 (CRL-1573) cells, were purchased from the American Type Culture Collection (ATCC, Manassas, VA, USA). The cells were cultured in RPMI-1640 medium (Gibco, Waltham, MA, USA), supplemented with 10% heat-inactivated fetal bovine serum, Cat # A5209501 (Thermo Fisher Scientific, Waltham, MA, USA), and 1% penicillin-streptomycin, Cat # 15140122 (Thermo Fisher Scientific, Waltham, MA, USA). All cells were incubated at 37 °C in a humidified incubator containing 5% CO_2_. Antibodies and reagents for immunoblotting, including PP2Ac (Catalog # 2038), AKT (Catalog # 9272), pAKT (S473) (Catalog # 9271), SRC (Catalog # 2108), pSRC (Catalog # 2101), GSK-3β (Catalog # 9315), pGSK-3β (Catalog # 9336), β-actin (Catalog # 3700 and Catalog # 8457), and okadaic acid (Catalog # 5934), were purchased from Cell Signaling Technology (Danvers, MA, USA). Anti-CIN85 antibody (Catalog # MAB8877) was purchased from R&D Systems (Minneapolis, MN, USA). FTY720 (Catalog # SML0700) and anti-rabbit and anti-mouse secondary antibodies (Catalog # 21537 and 21538M) were purchased from Sigma-Aldrich (St. Louis, MO, USA). Protease inhibitor cocktail (Catalog # P8340) was purchased from Millipore Sigma (Burlington, MA, USA).

### 2.4. Real-Time PCR (RT-PCR)

We used an RNeasy Mini Kit (Catalog # 74104, Qiagen, Germantown, MD, USA) to extract total RNA from leukemia cell lines and control HEK-293 cells. A QuantiTect Reverse Transcription Kit (Catalog # 205313, Qiagen, Germantown, MD, USA) was used to transcribe RNA into single-stranded cDNA. Real-time PCR was performed to detect expression of PP2Ac and CIN85 genes. Primer sequences for CIN85, PP2Ac, and other genes tested are shown in Table 1.

To ensure that the primer sets delivered accurate, specific, and precise data, they were validated before use. Validation of PCR primers was determined from a standard curve generated by serial dilutions of the template, and optimal efficiency was obtained at 100%, ensuring the specificity of the designed primer sets and product. To safeguard against random pipetting errors and well-to-well thermal variations, each reaction was routinely run in triplicate.

mRNA expression levels were quantified using RT^2^ SYBR Green qPCR Master Mix (Catalog # 330502, Qiagen, MD, USA) and detected using the CFX96 real-time PCR detection system (Bio-Rad, Hercules, CA, USA). Data analysis was performed using CFX Manager software, ver. 3.1 (Bio-Rad, Hercules, CA, USA). Data were analyzed by calculating 2^−ΔΔCt^ values and expressed as fold-change expression, compared with relative expression from HEK-293 cells, used as the experimental control, and from GAPDH, used as the internal control. Experiments were repeated three times using leukemia cells isolated from five different cultures.

### 2.5. siRNA Transfection

To knock down CIN85 expression, we used predesigned Silencer Select siRNA (5′-GACUGUUACCAUAUCCCAAtt-3′; Life Technologies, Waltham, MA, USA), and non-targeting Silencer Select Negative Control No. 1 siRNA (Life Technologies, Waltham, MA, USA) as a non-targeting siRNA control, using FuGENE^®^ SI RNA Transfection Reagent, Catalog # SI-1000 (Promega, Madison, WI, USA), according to the manufacturer’s recommendations. Briefly, K-562 cells in suspension were centrifuged for 5 min at 300× *g*. The cell pellet was resuspended at 2.5 × 10^5^ cells in 500 µL of complete medium (without antibiotics) and added to a 24-well plate. The cells were incubated at 37 °C with 5% CO_2_. In addition, 1 µL of FuGENE^®^ SI reagent was added to 50 µL of serum-free RPMI medium, mixed gently, and incubated at RT for 10 min. CIN85 siRNA at final concentrations of 25 nM and 50 nM was added to the diluted reagent, mixed thoroughly, and incubated for 20 min at RT to allow complex formation. The complex mixture was added to the cells in the 24-well plate, which was swirled gently to mix the contents with the cells. The cells were incubated for 72 h before harvesting to assess gene knockdown by immunoblotting.

### 2.6. PP2A Phosphatase Assay

The PP2A phosphatase assay was performed using a commercially available assay kit (Catalog # 17-313, Sigma-Aldrich, St. Louis, MO, USA). Briefly, plates were coated with 0.5 µg/mL protein lysate isolated from leukemia cells treated with siRNA targeting CIN85 at concentrations of 25 nM and 50 nM. The wells were blocked with 3% BSA and incubated at 37 °C for 2 h. After washing three times with TBS, the wells were incubated with phosphatase assay buffer and the phosphopeptide K R p T I R R (a PP2A-specific substrate) for 20 min at 30 °C, according to the manufacturer’s instructions. Phosphates released after dephosphorylation of the phosphopeptides were detected with malachite green and measured at 620 nm. The absorbance value was compared with a standard curve generated using the phosphate standard solution from the assay kit and expressed as pmol phosphate/min. Final results were compared with those from untreated controls.

### 2.7. Immunoblotting

K-562 and THP-1 cells were centrifuged, washed with PBS, and lysed in 100 µL of cell lysis buffer (Catalog # 9803, Cell Signaling Technology, MA, USA) containing protease and phosphatase inhibitors. The cell suspension was centrifuged at 15,000 rpm for 5 min at 4 °C, and the supernatant was collected and stored at −80 °C for further use. Protein concentration was estimated using the Bradford assay. To 30 µg of lysate, an equal volume (*v*/*v*) of 2× Laemmli sample buffer (Catalog # 1610737, Bio-Rad, CA, USA) was added. Samples were denatured at 99 °C for 10 min before being loaded onto a 10% SDS-PAGE gel. A Mini Trans-Blot System (Bio-Rad, Hercules, CA, USA) was used to transfer resolved proteins onto a nitrocellulose membrane. Non-specific binding was blocked with Tris-buffered saline containing 0.1% (*v*/*v*) Tween 20 (TBS-T) and 5% non-fat dry milk for 30 min at RT. Primary antibodies were diluted 1:1000 in TBS-T containing 5% skimmed milk or BSA, according to the manufacturer’s recommendations. Membranes were incubated overnight at 4 °C with continuous shaking. Membranes were washed three times with TBS-T and incubated with horseradish peroxidase (HRP)-conjugated secondary antibodies at 1:3000 dilution for 2 h at room temperature. After washing three times with TBS-T, bands were visualized using SuperSignal™ West Pico (Catalog # 34577) or West Femto Chemiluminescent Substrate (Catalog # 34096, Thermo Fisher Scientific, Waltham, MA, USA) in a ChemiDoc^®^ visualization system (Bio-Rad, Hercules, CA, USA). Band density was analyzed using image-analysis software, Image Lab, ver. 5.1 (Bio-Rad, Hercules, CA, USA). Bands from test samples were normalized to β-actin as a loading control. Experiments were repeated at least three times to avoid experimental bias.

### 2.8. MTS Assay

Proliferation of K-562 and THP-1 cells treated with CIN85 siRNA and untreated controls was measured with an MTS kit (CellTiter 96 Aqueous Non-Radioactive Cell Proliferation Assay, Cat# G5421, Promega, Walldorf, Germany). K-562 and THP-1 cells were cultured with CIN85 siRNA at concentrations of 25 nM and 50 nM. All cultures were performed in culture medium as described above. K-562 and THP-1 cell proliferation was then assessed after 72 h of treatment using an MTS kit as instructed by the manufacturer. Briefly, cells were incubated in MTS solution for 4 h at 37 °C, and absorbance was recorded at 490 nm using an ELISA plate reader (Spectra MR, Dynex Technologies, Denkendorf, Germany). Results from triplicate samples are presented as mean ± standard error.

### 2.9. Statistical Analysis

All statistical analyses were performed using GraphPad Prism Ver. 8.0 (GraphPad Software, La Jolla, CA, USA) to analyze the data statistically. To compare the means of two groups, a paired *t*-test was used, and for continuous dependent variables across three or more independent groups defined by a single categorical factor, a one-way analysis of variance (ANOVA) was employed. For experimental designs incorporating two distinct categorical independent variables, a two-way ANOVA was conducted to evaluate both the main effects of each factor and their interaction effect on the outcome measure. Following a significant omnibus F-test, post-hoc pairwise comparisons were performed using Dunnett’s multiple comparisons test and Tukey’s Honestly Significant Difference (HSD) test to control for multiple comparisons and preserve the family-wise error rate. Statistical significance was set at a two-tailed alpha level of *p* < 0.05 for all tests. Data are presented as mean ± standard error of the mean. All experiments were repeated at least three times (*n* = 3) representing three independent experiments, unless otherwise specified.

## 3. Results

### 3.1. Identification of Differentially Expressed Phosphatases (DEPs) and Adaptor Proteins in Leukemia Patient Datasets

**Figure 1:**
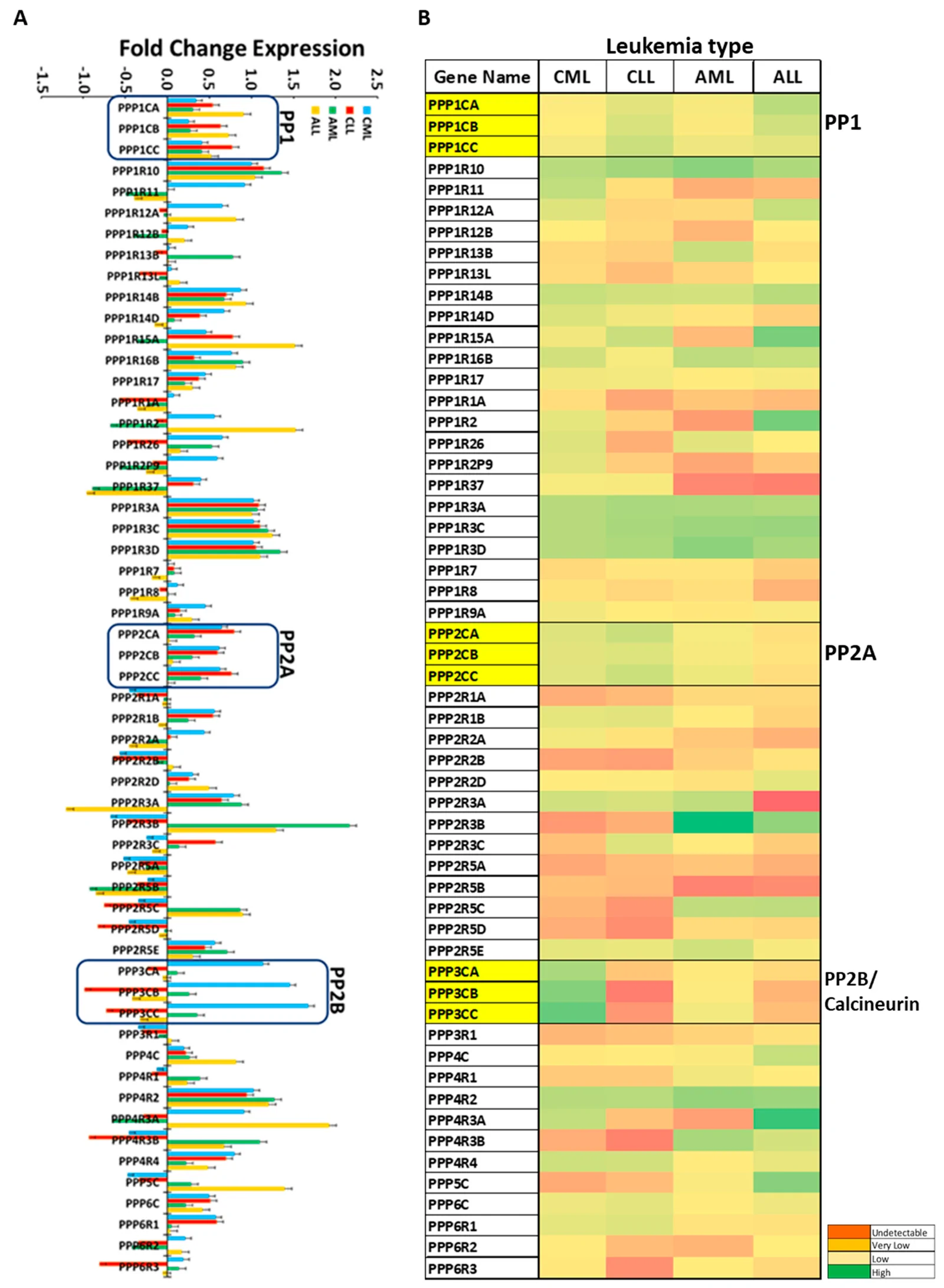

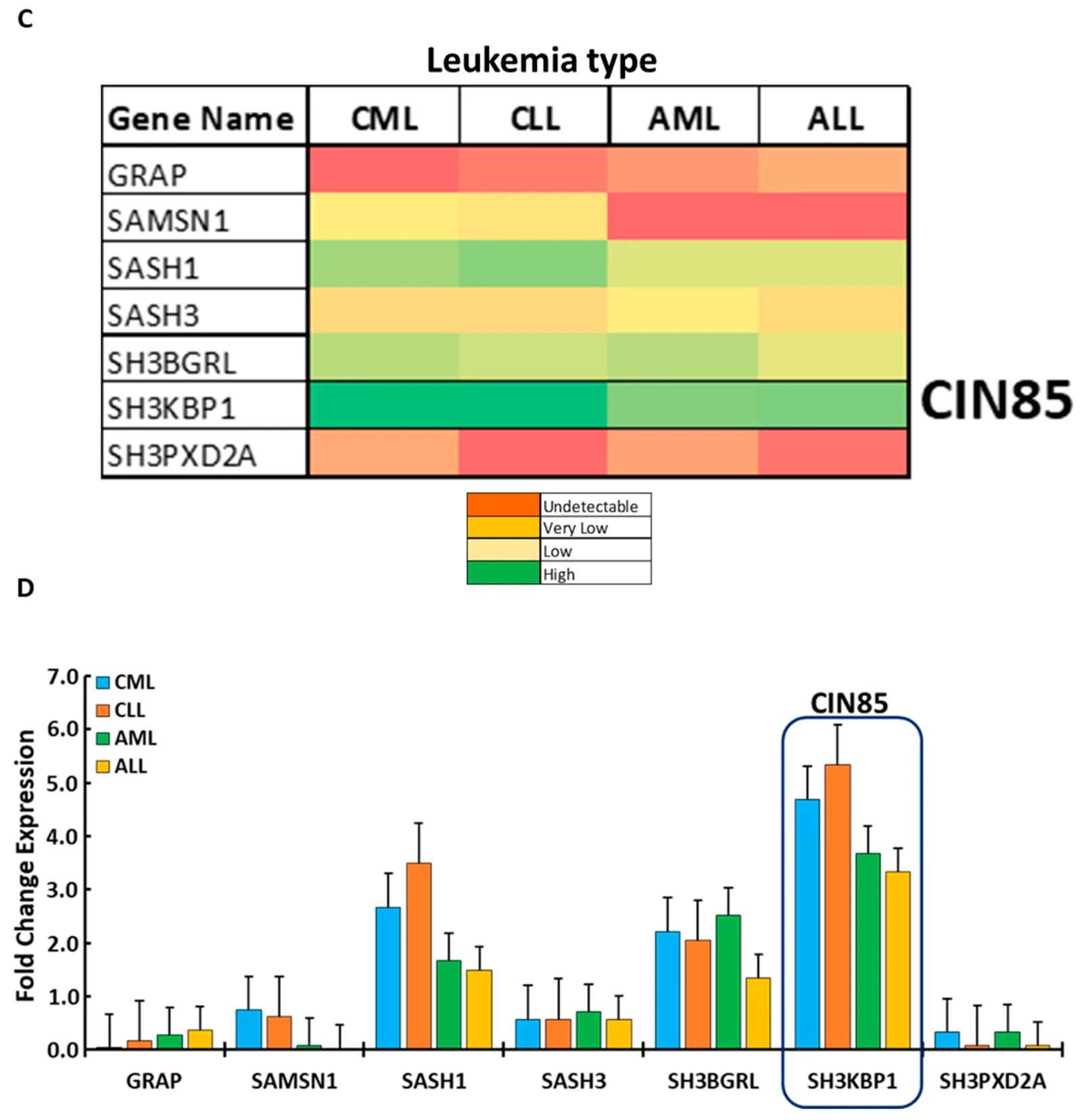
Expression profiles of Ser/Thr protein phosphatases and adaptor molecules in human leukemia datasets (GEO repository; patient versus healthy control blood/bone marrow samples). (**A**). Fold changes of differentially expressed genes (DEGs) between leukemia and control groups, with major Ser/Thr phosphatases boxed. (**B**). Heatmap of total phosphatase expression normalized to disease-free controls across multiple datasets, with important Ser/Thr phosphatases highlighted in boxes. (**C**). Heatmap of selected adaptor molecule expression normalized to disease-free controls, with *SH3KBP1 (CIN85)* boxed across datasets. (**D**). Fold changes of selected differentially expressed adaptor genes between leukemia and control groups.

We analyzed several transcriptomic datasets, including three for CML, two for AML, two for CLL, and one for ALL, to identify DEPs between leukemia patients and healthy individuals. A total of 57 phosphatases and 7 adaptor molecules with differential expression profiles were identified. The bar graph and heat map revealed significant expressional differences in phosphatase profiles between different leukemia’s (AML, CML, CLL and ALL), tested as compared to control (Figure 1A,B). Subsequently, transcriptomic data for adaptor molecules from patients with AML, CML, CLL, and ALL resulted in the identification of seven differentially expressed adaptor molecules. These results are presented as heat maps and bar graphs, as shown in Figure 1C,D.

Expression pattern analysis of major phosphatases showed that Protein Phosphatase 1 (PP1) was significantly reduced in all leukemias tested. However, in CML and AML patients, *PP1* was almost undetected for all subunits, including *PP1ca*, *PP1cb*, and *PP1cc*, as compared to CLL and ALL patients. Although not at significant levels, *PP2A* expression with all its subunits was detected in CML and CLL patients. Meanwhile, its expression was significantly reduced in AML patients and almost undetectable in ALL patients for all its subunits. In contrast, all subunits of *PP2B* were found to be significantly expressed in CML patients and expressed at significantly lower levels in AML patients. Expression of *PP2B* with all its subunits was almost undetectable in CLL and ALL patients, as shown in Figure 1A,B.

Expression profiles of several adaptor molecules showed differential expression, with several of them including nearly undetectable *GRAP*, *SAMSN1*, and *SH3PXD2A*. The most visible difference in expression profile was observed for *SH3KBP1 (CIN85)*, which was observed to be expressed at significantly higher levels in both CML and CLL patients as compared to the AML and ALL patients and compared to controls (Figure 1C,D).

### 3.2. Protein Phosphatase Expression Is Diminished in Human Leukemia Cell Lines

As observed in the expression profiles of hematologic tumors, the expression of almost all the prominent protein phosphatases, including *PP1* and *PP2A*, as well as *PP2B*, was observed to be downregulated. Contrarily, the expression of several adaptor molecules, including *CIN85*, was observed to be expressed at higher levels. In order to understand the role of differential expression profile of *PP2Ac* and *CIN85* in hematologic tumorigenesis, we first evaluated the expression pattern of *PP2Ac* and *CIN85*, along with a few known downstream targets in human leukemia cell lines.

**Figure 2:**
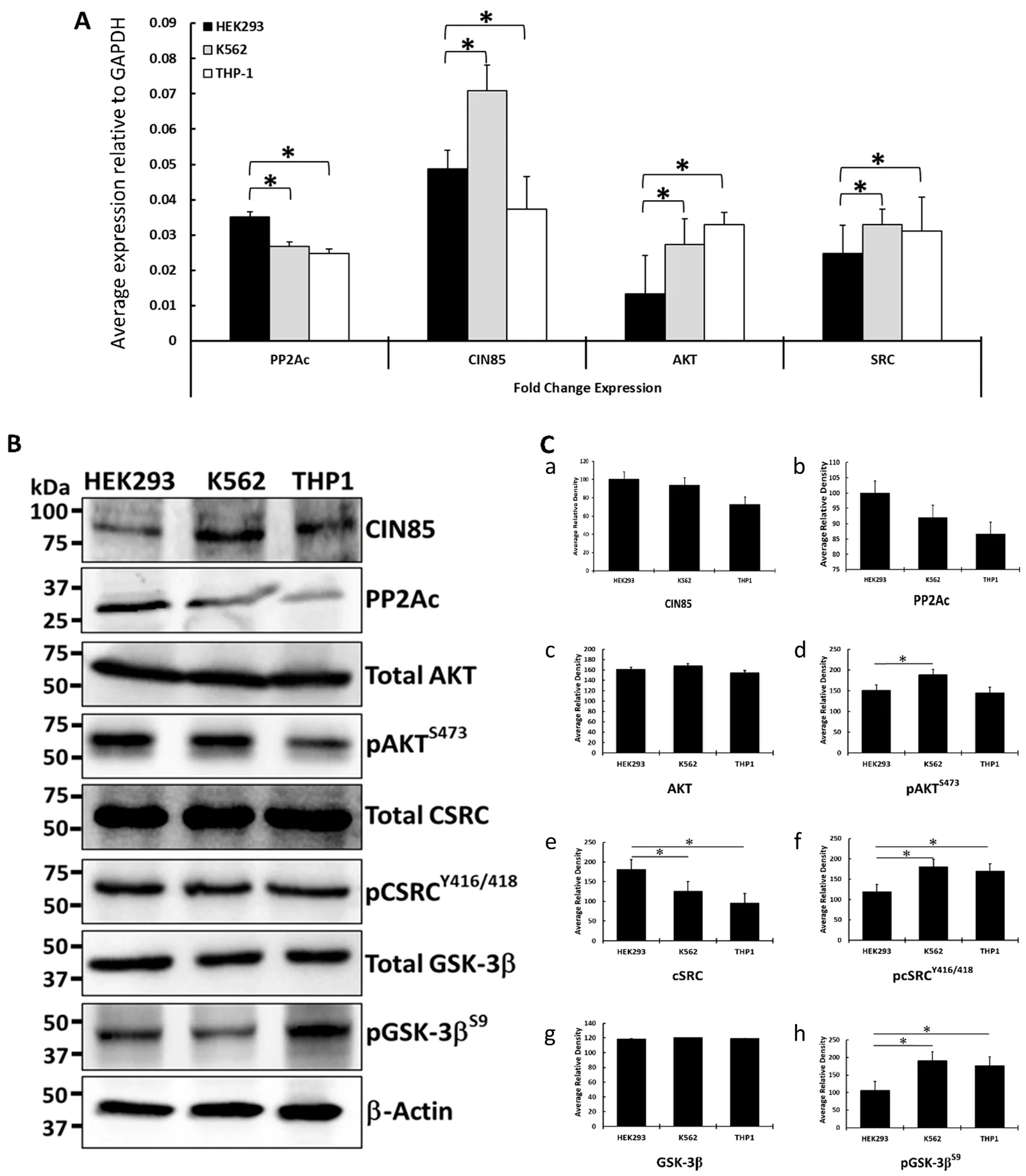
Diminished PP2Ac and high CIN85 expression in human leukemia cell lines, corroborating patient dataset findings. (**A**). mRNA expression of *PP2Ac*, *CIN85*, *AKT*, and *SRC* was analyzed using specific primers in leukemia cell lines with HEK293 as control. (**B**). Immunoblots show expression of *PP2Ac*, *CIN85*, and downstream targets (*AKT*, *SRC*, *GSK-3β*), along with their activation levels in K562 and THP1 cells versus HEK293 controls. (**C**). Densitometric quantification of immunoblot bands (Image Lab^®^). All experiments were repeated at least three times (*n* = 3), which represented three independent experiments, unless otherwise specified. Data are means ± SE; * *p* < 0.05.

Primers, as shown in Table 1, were designed based on the mRNA sequences of *PP2Ac*, *CIN85*, *AKT*, and *SRC* genes to analyze their mRNA expression in the human leukemia cell lines and HEK293 cells used as the control. As shown in Figure 2A, and as compared to the expression levels in HEK293 cells, the *PP2Ac* expression level was significantly decreased in both the K562 and THP1 cells. In comparison, a significant increase in *CIN85* expression was observed in K562 cells as compared to THP1 and the control HEK293 cells. *AKT* and *SRC* levels were significantly increased in both K562 and THP1 cells as compared to HEK293 cells.

In order to relate the expression pattern of PP2Ac and its associated genes at the proteomic level, we investigated whether the expression of total proteins of the phosphatases, the adaptor molecules, and the phosphorylation status of a few downstream target molecules were impacted in human leukemia cells. As shown in Figure 2B, CIN85 is significantly expressed in K562 cells, as compared to THP1 cells. However, equal levels of expression were observed in HEK293 cells (Figure 2B,Ca). As expected, PP2Ac mRNA levels were significantly reduced in K562 and THP1 cells, replicating the patient datasets, as compared to the HEK293 cells (Figure 2B,Cb). Although basal expression levels of the downstream target of PP2Ac and AKT were in equitable levels across all the cell lines tested, its phosphorylation was significantly reduced in THP1 cells, with no significant changes in K562 cells as compared to the control (Figure 2B,Cc,Cd). Total SRC levels were reduced in both the leukemia cell lines as compared to the HEK293 cells. However, phosphorylated SRC levels were significantly higher in both the leukemia cell lines as compared to the HEK293 cells (Figure 2B,Ce,Cf). Interestingly, the basal expression levels of GSK-3β were comparable in all the cells tested, yet significant expression levels in phosphorylated GSK-3β at Ser^9^ were observed in both K562 and THP1 cells as compared to the HEK293 cells (Figure 2B,Cg,Ch).

### 3.3. Activation of PP2Ac Modulates Phosphorylation of Its Downstream Kinase Targets

**Figure 3:**
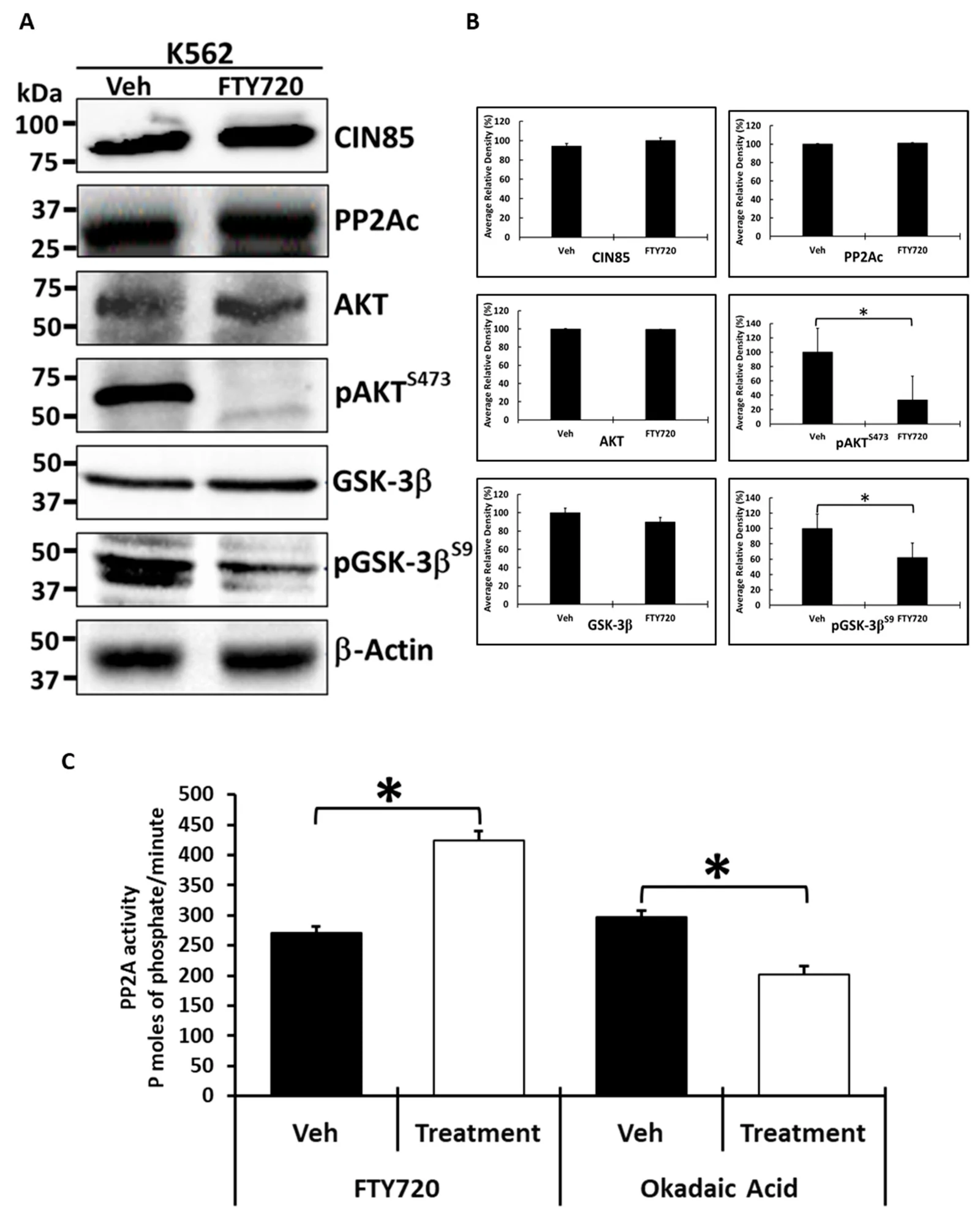
PP2Ac activation modulates phosphorylation of downstream kinase targets. (**A**). Immunoblots of K562 lysates treated with FTY720, showing phosphorylation of AKT, SRC, andGSK-3β. (**B**). Densitometric quantification of immunoblot bands (Image Lab^®^). (**C**). PP2A phosphatase activity assay in K562 cells treated with FTY720 (activator) and OA (inhibitor). All experiments were repeated at least three times (*n* = 3), which represented three independent experiments, unless otherwise specified. Data are means ± SE; * *p* < 0.05.

In order to establish a model to test the impact of CIN85 knockdown, we first studied the impact of PP2Ac activation on the functional consequence and its mechanism using the already known activators and inhibitors of PP2A on the leukemia cell line K562. We confirmed the effect of PP2Ac activation using the commercially available PP2Ac activator FTY720 and the inhibitor OA on the modulation of downstream serine/threonine kinase phosphorylation in K562 cells. Treatment with FTY720 did not affect the basal expression levels of CIN85, PP2Ac, total AKT, and GSK-3β; however, it abolished the elevated phosphorylation and activation of AKT and GSK-3β, as seen in the K562 cells, suggesting that this activator specifically impacts the phosphorylation of PP2Ac downstream effector kinases, regardless of CIN85 expression levels (Figure 3A,B). Next, we established the consequence of PP2A activator FTY720 and the PP2Ac inhibitor on phosphatase activity in the K562 cell line. As shown in Figure 3C, a significant increase in PP2A phosphatase activity was observed in K562 cells treated with FTY720, whereas a significant decrease in PP2A phosphatase activity was observed in cells treated with OA, confirming the outcome of PP2Ac activators on both the phosphatase activity and its impact on the phosphorylation status of the downstream serine/threonine kinases.

### 3.4. Inhibition of CIN85 Downregulates PP2Ac-Mediated Phosphorylation of Its Downstream Kinase Targets

**Figure 4:**
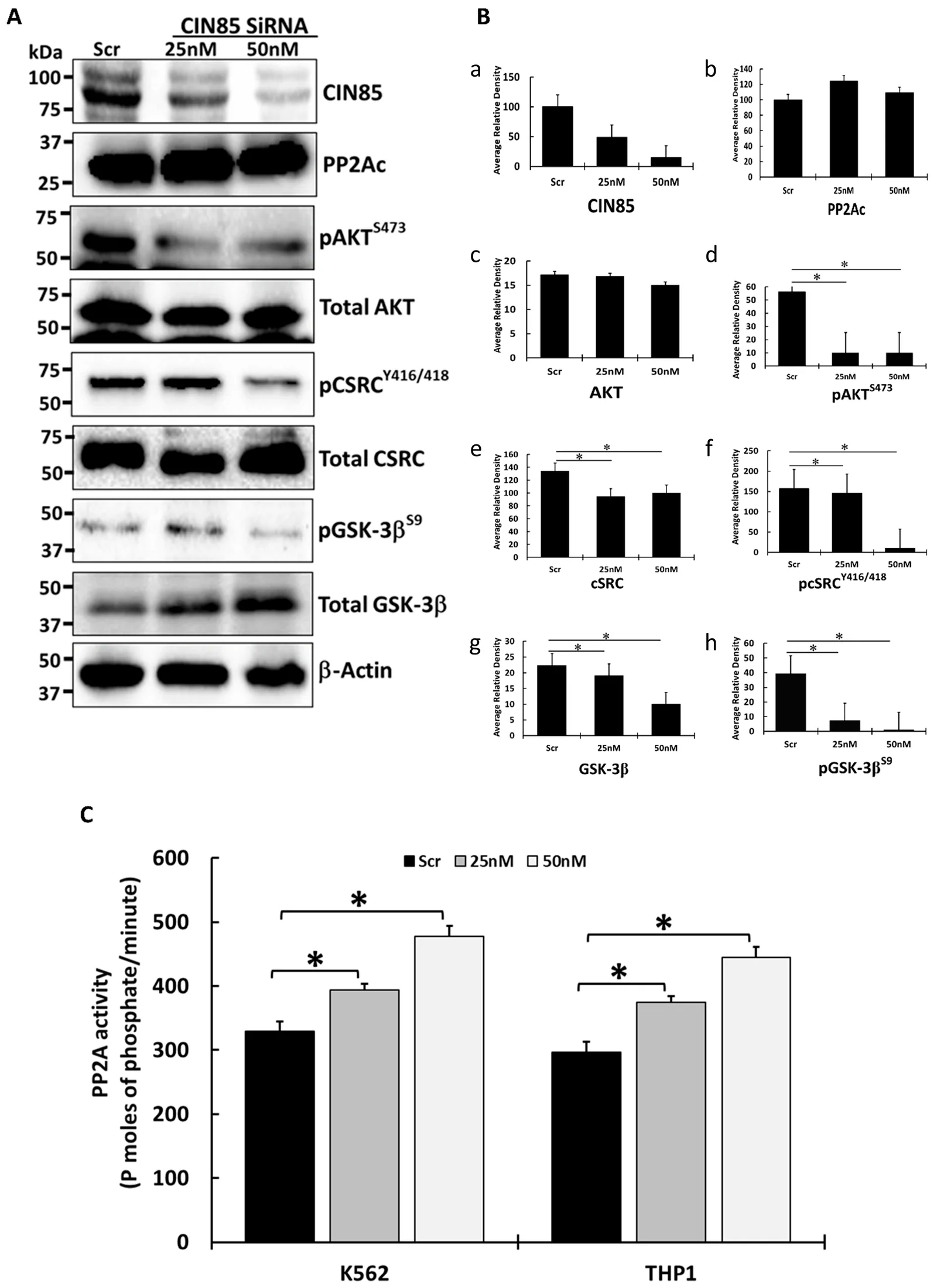
siRNA-mediated CIN85 knockdown reduces PP2Ac-dependent phosphorylation of downstream kinase targets. K562 cells were transfected with 25 nM and 50 nM of siCIN85 or scrambled control (SCR). (**A**). After 48 h, whole-cell lysates were immunoblotted for CIN85, PP2Ac, and its downstream effectors. (**B**). Densitometric quantification of immunoblot bands (Image Lab^®^). (**C**). PP2A phosphatase activity was measured in K562 and THP1 cells treated similarly, using a commercial activity kit. All experiments were repeated at least three times (*n* = 3) that represented three independent experiments, unless otherwise specified. Data are means ± SE; * *p* < 0.05.

As established earlier by us, the ability of CIN85 to associate with PP2Ac to form a CIN85-PP2Ac complex modulates the phosphorylation of several downstream kinases. To test the contribution of CIN85 in restraining PP2Ac activity in leukemia cell lines, we transfected the leukemia cell line K562 with CIN85 siRNA to knock down the protein expression, using scrambled siRNA as the control (Figure 4A). We first determined the status of CIN85 knockdown by the specific siRNA, and as shown in Figure 4Ba, cells transfected with 25 nM and 50 nM siRNA reduced the expression levels of CIN85 to 55% and 19%, respectively, as compared to the SCR control. As expected, CIN85 siRNA did not change the expression levels of the PP2Ac protein (Figure 4A,Bb). We next assessed the impact of CIN85 knockdown on the phosphorylation status of the downstream kinase targets of PP2Ac. As shown in Figure 4A,Bc, CIN85 knockdown significantly reduced the phosphorylation of AKT at Ser^473^ at both 25 nM and 50 nM concentrations as compared to the scrambled control. However, no change in the expression levels of basal AKT was observed in the siRNA treated or SCR control cells (Figure 4A,Bd). Additionally, 50 nM of CIN85 siRNA significantly reduced the phosphorylation of SRC at Tyr^416/418^, as compared to the 25 nM of siRNA and the scrambled control, with no change in the basal SRC expression (Figure 4A,Be,f). Correspondingly, both 25 nM and 50 nM of CIN85 siRNA concentrations reduced the GSK-3β phosphorylation at Ser^9^, which is significant compared to the scrambled control (Figure 4A,Bg). As expected, no significant change in the total GSK-3β levels was observed in any of the siRNA concentrations tested, as well as in the scrambled control (Figure 4A,Bh).

Next, we investigated if CIN85 knockdown influenced the phosphatase activity of PP2Ac that may have led to modulation in the phosphorylation of downstream targets. Leukemia cell lines K562 and THP1 were treated with 25 nM and 50 nM of CIN85 siRNA and scrambled control siRNA, and the resultant PP2A phosphatase activity was assayed using a PP2A phosphatase activity kit. As shown in Figure 4C, a significant increase in PP2A phosphatase activity was observed in both the cell lines, after knocking down of CIN85, in a dose-dependent manner as compared to the scrambled control.

### 3.5. Modulation of PP2Ac Activity Through CIN85 Inhibition Influences the Functional Consequences of the Leukemia Cells

**Figure 5:**
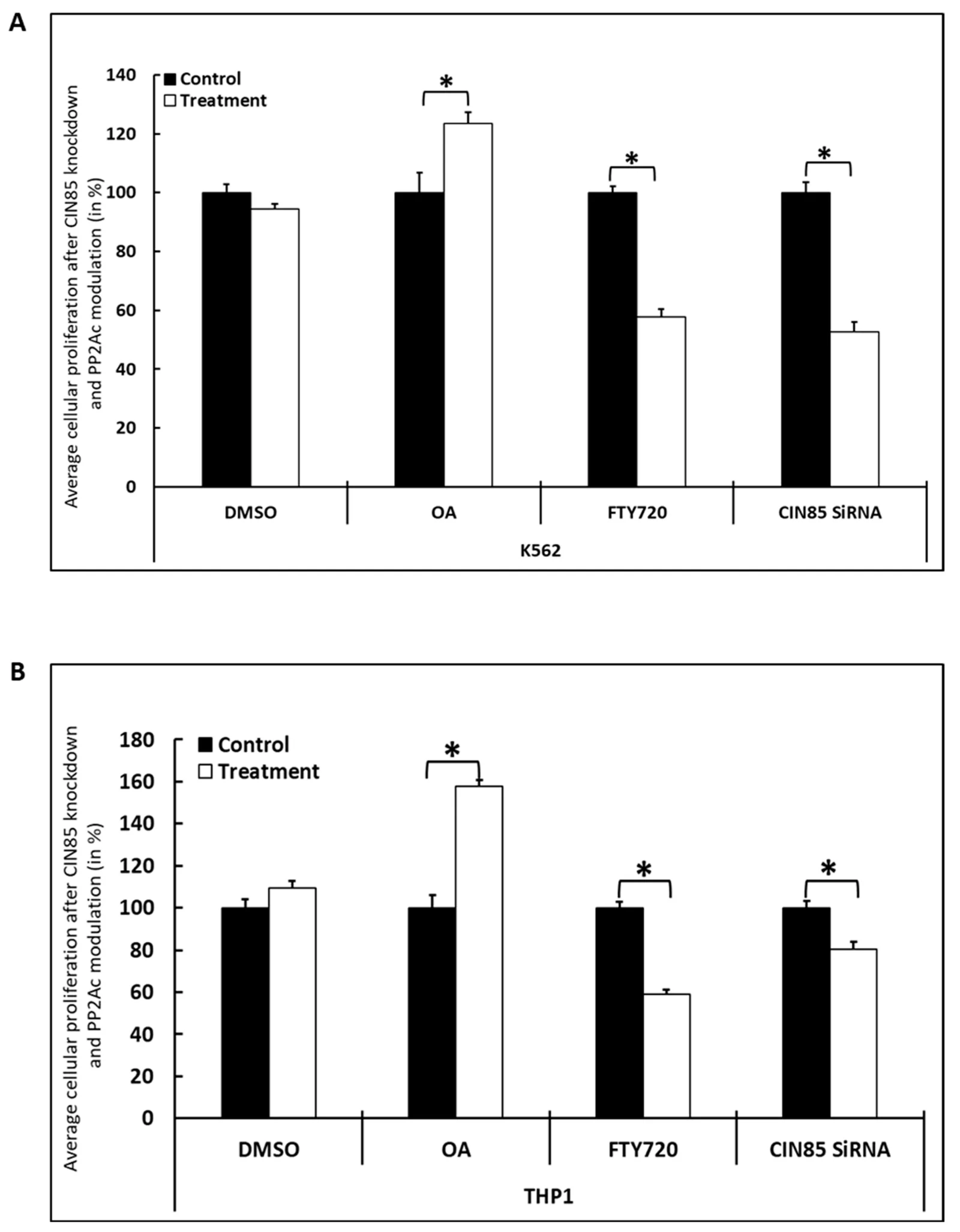
CIN85 inhibition modulates leukemia cell proliferation, evaluated by MTS assay. (**A**). K562 proliferation decreased significantly with siCIN85, similar to FTY720 treatment, and differed from OA, DMSO, and untreated controls. (**B**). THP1 cells exhibited analogous effects, with siCIN85 reducing proliferation to levels comparable to FTY720, relative to OA or DMSO controls. All experiments were repeated at least three times (*n* = 3), which represented three independent experiments, unless otherwise specified. Bars show mean proliferation percentages ± SE; * *p* < 0.05.

Finally, we investigated the effect of PP2Ac activation through downregulation of CIN85 on the functional consequences of leukemia cell lines K562 and THP1 (Figure 5). As expected, in the presence of OA, which inhibits PP2Ac activity, both the K562 and THP1 cells showed about 25% and 60% increases in cellular viability, respectively, as compared to untreated control cells (Figure 5A,B). Contrarily, the cell lines K562 and THP1 treated with FTY720, which activates PP2Ac, showed about 40% and 35% decreases in cellular proliferation, as compared to untreated controls (Figure 5A,B). Interestingly, both K562 and THP1 cells treated with 50nM of CIN85 siRNA, which knocks down its expression and releases PP2Ac from the PP2Ac-CIN85 complex, showed a significant decrease (60% and 30%, respectively) in cellular proliferation, as shown in Figure 5A,B. These findings suggest that disruption of the PP2Ac-CIN85 complex may lead to detrimental cellular consequences for both K562 and THP1 cells.

## 4. Discussion

Protein phosphorylation and dephosphorylation constitute one of the most fundamental regulatory mechanisms governing cellular signaling, proliferation, differentiation, and survival [21]. Maintenance of this dynamic equilibrium depends on the coordinated actions of protein kinases and phosphatases, whose opposing activities ensure precise regulation of intracellular signaling networks [2]. Although constitutive activation of oncogenic kinases has long been recognized as a hallmark of leukemia, increasing evidence indicates that functional impairment of tumor-suppressive phosphatases contributes equally to leukemogenesis. Among these phosphatases, PP2A has emerged as one of the most important tumor suppressors in hematological malignancies [22]. However, despite extensive studies demonstrating PP2A inactivation in leukemia, the molecular mechanisms responsible for suppressing its phosphatase activity remain incompletely understood. While endogenous PP2A inhibitors, including SET, CIP2A, PME-1, and ARPP19, have been extensively investigated, the contribution of adaptor proteins to PP2A regulation has received considerably less attention [23,24,25]. In particular, the biological significance of the PP2Ac-CIN85 interaction in leukemia has remained largely unexplored. In the present study, we demonstrate that disruption of the PP2Ac-CIN85 complex restores PP2A phosphatase activity, attenuates oncogenic kinase signaling, and suppresses leukemia cell proliferation, thereby identifying CIN85 as a previously underappreciated regulator of PP2A function and a potential therapeutic target in hematological malignancies.

Our bioinformatics analyses revealed broad alterations in phosphatase expression across AML, CML, CLL, and ALL patient samples, suggesting that phosphatase dysregulation may represent a common molecular feature of leukemia. Several studies have demonstrated that reduced phosphatase activity creates a permissive environment for sustained oncogenic signaling [26]. Protein phosphatases, particularly PP1 and PP2A, function as negative regulators of numerous signaling cascades that control cell-cycle progression and apoptosis [26,27]. The observed reduction in PP1 family members across leukemia subtypes is noteworthy because PP1 participates in chromosome segregation, DNA repair, and checkpoint regulation. Previous studies have demonstrated that disruption of PP1-mediated regulation of chromosome segregation, DNA damage repair, and mitotic checkpoint signaling promotes chromosomal instability and uncontrolled cellular proliferation, thereby creating a permissive environment for leukemogenesis and disease progression [28,29]. Therefore, the collective reduction of both PP1 and PP2A family members may synergistically amplify oncogenic signaling in leukemic cells.

Among the dysregulated phosphatases identified in our transcriptomic analysis, *PP2A* emerged as one of the most consistently altered tumor suppressors across leukemia subtypes, underscoring its central role in maintaining hematopoietic homeostasis. PP2A is a highly conserved serine/threonine phosphatase that negatively regulates numerous oncogenic signaling pathways by dephosphorylating key substrates, including AKT, c-MYC, BCR-ABL, ERK, STAT3, SRC, and β-catenin, thereby controlling cellular proliferation, survival, differentiation, and self-renewal [9]. Functional impairment of PP2A has been widely implicated in the pathogenesis of both myeloid and lymphoid malignancies, where sustained suppression of its phosphatase activity permits persistent activation of pro-survival signaling networks and promotes therapeutic resistance [6,7]. Interestingly, although our transcriptomic analyses demonstrated reduced *PP2A* expression in leukemia patient samples, PP2Ac protein remained detectable in leukemia cell lines. This apparent discrepancy between mRNA and protein abundance has been reported previously and may arise from post-transcriptional regulation, altered protein turnover, compensatory translational mechanisms, or differential assembly of the PP2A holoenzyme [9,30]. Importantly, accumulating evidence indicates that functional inhibition rather than reduced expression is the predominant mechanism underlying PP2A inactivation in leukemia. Indeed, leukemic cells frequently retain measurable PP2Ac protein but suppress its catalytic activity through endogenous inhibitory molecules, including SET, CIP2A, PME-1, and ARPP19, highlighting that preservation of PP2Ac expression does not necessarily reflect preservation of phosphatase function [7,10,24,31].

A particularly significant finding of this study is the elevated expression of CIN85 in leukemia patient samples and leukemia cell lines. CIN85 is increasingly recognized as a multifunctional adaptor protein that orchestrates diverse protein–protein interactions involved in receptor trafficking, cytoskeletal remodeling, intracellular signal transduction, and immune receptor signaling [15]. Through its multiple SH3 domains, CIN85 assembles signaling complexes that regulate receptor endocytosis, signal propagation, and cytoskeletal dynamics, thereby influencing numerous physiological and pathological processes, including tumor progression. Although the role of CIN85 has been extensively characterized in solid tumors, its contribution to hematological malignancies remains poorly understood [32,33]. It has been demonstrated that CIN85 promotes tumor cell invasion and metastasis by modulating SRC-dependent signaling pathways [34]. Similarly, studies in multiple cancers have linked CIN85 overexpression with enhanced cellular proliferation, migration, and poor clinical outcomes [16,35]. Although CIN85 has been less extensively investigated in hematological malignancies, the elevated expression observed in our study suggests that similar signaling mechanisms may contribute to leukemia progression.

The observed activation of AKT-, SRC-, and GSK-3β-signaling pathways in leukemia cells further supports impaired phosphatase-mediated regulation. The PI3K/AKT pathway is among the most frequently activated pathways in AML and CML and contributes to leukemic stem-cell survival, resistance to apoptosis, and therapeutic failure [36]. PP2Ac directly dephosphorylates AKT, thereby terminating AKT-mediated survival signaling [37]. Likewise, SRC family kinases are constitutively activated in several hematological malignancies and cooperate with BCR::ABL1 and FLT3 signaling to promote leukemic proliferation [38,39].

GSK-3β is a key downstream effector of the PI3K/AKT-signaling pathway and plays a context-dependent role in regulating cell proliferation, differentiation, and survival. The hyperactivation of AKT promotes inhibitory phosphorylation of GSK-3β at Ser^9^, resulting in suppression of its kinase activity and stabilization of β-catenin, thereby enhancing the transcription of genes involved in cell survival and proliferation [40,41]. Conversely, activation of PP2Ac antagonizes this oncogenic signaling by promoting dephosphorylation of AKT and its downstream substrates, including GSK-3β, thereby restoring GSK-3β activity and attenuating survival signaling [42]. In the present study, disruption of the PP2Ac-CIN85 complex significantly reduced phosphorylation of GSK-3β (Ser^9^), indicating restoration of PP2A phosphatase activity and suppression of AKT-dependent oncogenic signaling. These findings further support the role of PP2A as a central negative regulator of leukemia-associated signaling networks.

The restoration of PP2A phosphatase activity following disruption of the PP2Ac-CIN85 complex is consistent with growing evidence supporting PP2A reactivation as a promising therapeutic strategy for hematological malignancies. Pharmacological PADs restore PP2A tumor-suppressive function by promoting dephosphorylation of multiple oncogenic substrates and simultaneously suppressing leukemia-associated signaling pathways. Among these, FTY720 (Fingolimod) was the first PAD shown to restore PP2A activity, inhibit BCR::ABL1 and AKT signaling, and induce apoptosis in tyrosine kinase inhibitor-resistant CML cells while sparing normal hematopoietic progenitors [1,43]. However, it is noteworthy that FTY720 is a multi-targeted drug whose diverse biological effects cannot be attributed solely to PP2A activation. Its PP2A-independent activities include the induction of non-apoptotic cell death, ROS generation, direct enzyme inhibition, and metabolic interference, which are crucial to its therapeutic profile and contribute to its efficacy in various disease models, including cancer.

More recently, next-generation PADs, including DT-061 and other selective PP2A holoenzyme stabilizers such as DBK-1154 and ATUX-792, have demonstrated encouraging preclinical antitumor activity by restoring PP2A function and overcoming resistance to kinase-targeted therapies [7,44]. These findings, together with our results, highlight PP2Ac-CIN85 disruption as a potential therapeutic approach for restoring PP2A activity in leukemia.

Perhaps the novel aspect of the present study is the demonstration that CIN85 functions as a negative regulator of PP2Ac activity in leukemia cells. Our previous study first identified a direct physical interaction between the PP2Ac and the adaptor protein CIN85 and demonstrated that CIN85 binding suppresses PP2A phosphatase activity [19]. The current findings extend these observations by demonstrating that disruption of CIN85 expression enhances PP2A phosphatase activity and attenuates phosphorylation of multiple downstream oncogenic kinases. This finding is particularly important because most currently known mechanisms of PP2A inhibition involve SET, CIP2A, or PME-1. CIN85 may therefore represent a previously underappreciated regulatory component within the PP2Ac signaling network. Given the multifunctional scaffold properties of CIN85, it is plausible that CIN85 not only directly restrains PP2Ac activity but also facilitates the assembly of signaling complexes that spatially segregate PP2Ac from its substrates, thereby promoting sustained kinase activation. Future studies investigating the molecular architecture of the PP2Ac-CIN85 complex may reveal additional regulatory mechanisms contributing to leukemogenesis.

The functional consequences of CIN85 depletion observed in both K562 and THP1 cells further reinforce the biological significance of this interaction. Restoration of PP2A activity following CIN85 knockdown was associated with marked suppression of leukemia cell proliferation. These findings align with numerous studies demonstrating that PP2Ac reactivation induces cell-cycle arrest, promotes apoptosis, suppresses leukemic stem-cell maintenance, and enhances sensitivity to chemotherapeutic agents [1,12,45]. Importantly, because PP2A simultaneously regulates multiple signaling pathways, therapeutic approaches aimed at restoring PP2Ac activity may provide broader antitumor effects than strategies targeting individual kinases.

Despite these promising mechanistic insights garnered from our in vitro studies, several methodological considerations warrant acknowledgment. Firstly, the current investigation is exclusively cell-based, and the absence of in vivo animal models precludes any assessment of therapeutic efficacy. Secondly, the lack of validation using primary patient-derived leukemic specimens restricts the extrapolation of our findings to the heterogeneous clinical landscape. Collectively, addressing these limitations and incorporating them in future translational studies will establish the clinical viability and therapeutic safety of targeting this CIN85-PP2Ac interaction.

## 5. Conclusions

In conclusion, our findings strengthen the growing body of evidence supporting PP2A as a central tumor suppressor in leukemia and suggest CIN85 as a novel regulator of PP2Ac-mediated signaling. The elevated expression of CIN85, together with impaired PP2A activity, may contribute to persistent activation of AKT-, SRC-, and GSK-3β-dependent signaling pathways that support leukemic cell survival and proliferation. Disruption of the PP2Ac-CIN85 complex restores phosphatase activity, suppresses oncogenic signaling, and inhibits leukemia cell growth, highlighting a previously unrecognized therapeutic vulnerability. Targeting the PP2Ac-CIN85 interaction, either alone or in combination with established PP2A-activating drugs and kinase inhibitors, identifies CIN85 as a potential regulator of PP2A-associated signaling in leukemia and provides a rationale for further investigation of the CIN85-PP2A interface as a therapeutic target.

## Figures and Tables

**Table 1:** PCR Primers for RT-PCR analysis of PP2Ac and its target molecules.

Gene Name	Primer Name	Primer Sequence
PP2Ac	PP2A-F	TGGTGGTCTCTCGCCATCTA
	PP2A-R	TGTAACCAGCTCCTCGAGGA
CIN85	CIN85-F	GGTGGGAAGGAGAGCTGAAC
	CIN85-R	GATGACAGGAGCGGATGGAG
AKT	AKT1-F	GGACAAGGACGGGCACATTA
	AKT1-R	CGACCGCACATCATCTCGTA
c-SRC	c-SRC-F	ACATCCCCAGCAACTACGTG
	c-SRC-R	CAGTAGGCACCTTTCGTGGT
B-Actin	B-Actin-F	CACCATTGGCAATGAGCGGTTC
	B-Actin-R	AGGTCTTTGCGGATGTCCACGT

## Data Availability

All data and materials will be available upon request.

## Supplementary Materials

The following supporting information can be downloaded at: https://www.mdpi.com/article/10.3390/cells15201897/s1, Table S1: Description of Leukemia datasets.

## Abbreviations

The following abbreviations are used in this manuscript:

AML	Acute Myeloid Leukemia
ALL	Acute Lymphoblastic Leukemia
CML	Chronic Myeloid Leukemia
CLL	Chronic Lymphocytic Leukemia
PAD	PP2A-activating drugs
OA	Okadaic Acid
CIN85	C-cbl interacting protein of 85kDA
PP2Ac	Protein Phosphatase 2A (c-Subunit)
